# Bilateral iliopsoas intramuscular bleeding following anticoagulant therapy with heparin: a case report

**DOI:** 10.4076/1757-1626-2-7534

**Published:** 2009-07-23

**Authors:** Luigi Murena, Ettore Vulcano, Emanuela Salvato, Marco Marano, Fabio D’Angelo, Paolo Cherubino

**Affiliations:** Department of Orthopaedic and Trauma Surgery, Insubria UniversityViale Borri 57, 21100 VareseItaly

## Abstract

Iliopsoas haematoma is an uncommon complication that may arise during anticoagulant therapy, especially with heparin and warfarin. Besides determining patient distress secondary to femoral nerve compression, this event may progress to life-threatening complications and require expensive treatments. We describe the case of a 70-year-old healthy man complaining of severe bilateral groin, lumbar and thigh pain, and paralytic ileus after therapy with heparin. The angio-computed tomography scan observed bilateral iliopsoas haematomas. In view of the clinical and radiological scenarios, we ordered a diagnostic and therapeutic angiography of the bleeding vessels by trans-catheter arterial embolization of the fourth right lumbar artery trunk. The treatment proved to be beneficial from a clinical, radiological and laboratory point of view. To the best of our knowledge, this is the first reported case of bilateral iliopsoas haematoma occurring in a male treated with therapeutic levels of heparin alone.

## Introduction

Iliopsoas haematoma is an uncommon complication that may arise during anticoagulant therapy, especially with heparin and warfarin. Most cases of iliopsoas haematoma are unilateral, although it has been reported to be bilateral in few studies [1]. Besides determining patient distress secondary to femoral nerve compression, this event may progress to life-threatening complications and require expensive treatments [2]. The neurological clinical scenario arising from the haematoma may induce the physician to misdiagnose the real cause of the symptoms. Furthermore, postponing the diagnosis may indeed have devastating consequences on the patient outcome.

We describe the case of a patient complaining of severe bilateral groin, lumbar and thigh pain, and paralytic ileus. To the best of our knowledge, this is the first reported case of bilateral iliopsoas haematoma occurring in a male treated with therapeutic levels of heparin alone.

## Case presentation

A 70-year-old man, Italian Caucasian, with no underlying medical condition, was admitted to our orthopaedic surgery department for a circular blade injury. He presented a deep lesion of the anterior aspect of the right elbow, with complete section of the humeral artery, median and radial nerves, and radial capitellum fracture. He was urgently submitted to surgical reconstruction of the humeral artery with saphenous vein graft. At the same time, osteosynthesis of the radial capitellum was also perfomed, using three bioabsorbable nails (SmartNail, Conmed Linvatec, Largo, FL).

His preoperative laboratory exams were remarkable except for haemoglobin and platelet count, 10.2 g/dL and 119,000 per μl^3^ respectively. Post-operative laboratory exams showed decreased haemoglobin (7.4 g/dL). In view of the anaemia, we decided to transfuse the patient with two units of packed red blood cells. Post-transfusion haemoglobin increased to 9.7 g/dL.

Post-operative therapy was started with enoxaparin sodium 16,000 U/day. One week later, the patient underwent surgery for reconstruction of median and radial nerves with a sural nerve graft. Haemoglobin levels remained unchanged ever since the blood transfusion and platelet count returned within normal limits. However, the day after the nerve reconstruction, the patient presented with mild haemoglobin decrease (9 g/dL) and complained of right-side groin pain radiating to the anterior aspect of the right thigh. However, hip X-rays were negative. Within the following four days, haemoglobin levels and platelet count decreased to 8.3 g/dL and 115,000 per μl^3^ respectively, while pT, INR and APTT were within normal limits. We decided to transfuse the patient with two more packed red blood cells units. Moreover, the groin pain became bilateral and involved the low back and the posterior aspect of both thighs.

At physical examination, the patient was pale and presented with flexed hips, bilateral femoral nerve palsy more severe on the right, and normal leg and foot pulses. Palpation of the lumbar paraspinal muscles and spinous processes evoked pain, and Lasegue sign was positive. Furthermore, the patient’s abdomen was “quiet”, tense, bloated, and painful at deep palpation, suggestive of paralytic ileus. X-ray evaluation of the abdomen showed air-fluid levels.

**Figure 1.**
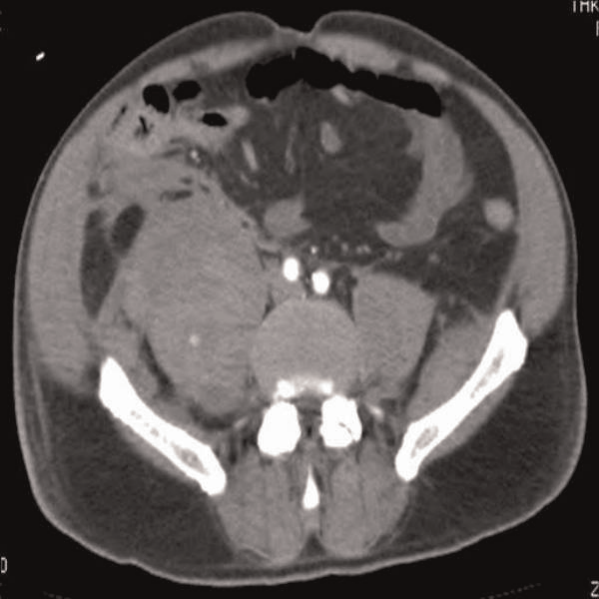
Abdominal angio-CT scan: bilateral iliopsoas haematomas.

Suspecting a myeloradiculitis we ordered a lumbar MRI, which was negative for nerve root compression. Nonetheless, his condition deteriorated over the following two days in terms of groin and lumbar pain, with haemoglobin values and platelet count down to 8.6 g/dL and 86,000 per μl^3^ respectively. Again, two packed red blood cells units were transfused. Further evaluation of the abdomen was obtained with an angio-CT scan, which revealed the presence of large iliopsoas haematomas bilaterally (right bigger than left) and suggested intramuscular active bleeding (Figure 1). We proceeded with immediate suspension of enoxaparin sodium. The following three days, the patient was transfused with a total of six packed red blood cells units (two per day) given a non-increasing haemoglobin value (range, 8.2 to 8.7 g/dL), and four units of fresh frozen plasma given the platelet count progressively decreasing to 39,000 per μl^3^.

In view of the clinical and radiological scenarios, we ordered a diagnostic and therapeutic angiography of the bleeding vessels by trans-catheter arterial embolization (TAE) of the fourth right lumbar artery trunk. The treatment proved to be beneficial, for the haemoglobin and platelet count progressively improved over the following days, despite an initial decrease of haemoglobin over the first three days which made two more packed red cell transfusions necessary. A CT scan obtained 48 hours after TAE showed bilateral iliopsoas haematomas of unchanged volume compared to the previous CT. Clinical, radiological and laboratory parameters all returned within normal limits over the following 20 days.

## Discussion

Iliopsoas haematoma is an infrequent complication of anticoagulant therapy. Although bleeding into the iliacus, psoas and iliopsoas muscles is usually unilateral [1], very few cases in the literature have reported bilateral iliopsoas haematomas [3-6]. From a clinical point of view, patients affected by iliopsoas haematoma may complain of lumbar or groin pain, lumbar plexus neuropathy or in more severe cases, can present with massive bleeding and hypovolemic shock [7]. Interestingly, as demonstrated in Table 1, our patient is the first reported case of bilateral iliopsoas bleeding in an otherwise healthy male treated with heparin alone.

**Table 1.** Cases of anticoagulant-induced bilateral iliopsoas haematomas reported in the literature

Author (year)	Age(yrs)/Sex	Indication for anticoagulation	Type of anticoagulant	Onset after administration of anticoagulant	Coagulation level	Treatment
Storen (1978)	55/F	Deep vein thrombosis	Heparin/warfarin	5 days	Therapeutic	Surgery
Barontini (1986)	65/F	Myocardial infarct	Antiaggregant (Teklid 2 cps) Anticoagulant (Calcipatina 0.5 × 2)	9 months 3 months	Normal	Conservative
Niakan (1991)	54/F	Pulmonary embolism	Heparin/warfarin	Unknown	Unknown	Right side surgery, Left side conservative
Jamjoom (1993)	19/F	Deep vein thrombosis	Heparin/warfarin	3 weeks	Therapeutic	Surgery
Wada (2005)	85/F	Transient ischemic attack	Heparin/warfarin	3 days	Therapeutic	Conservative/trans-catheter arterial embolization
Our Case	70/M	Neurovascular reconstruction	Heparin	19 days	Therapeutic	Right side TAE, Left side conservative

From the first day of admission to the orthopaedic surgery department, our patient presented with pT, INR and APTT values within the therapeutic range. This data is comparable to that reported in the literature with respect to both unilateral and bilateral iliopsoas haemorrhage [1,3-7].

Treatment of this uncommon complication of anticoagulant therapy is currently still controversial. In fact, while some authors have reported conservative treatment of iliopsoas bleeding, others submitted their patient to surgical evacuation or TAE [1,3-7]. As reported by Sasson *et al.*, conservative management was reserved to patients with mild-to-moderate femoral nerve palsy associated to inconspicuous bleeding, whereas patients with severe haemorrhage were submitted to surgical decompression [1]. Furthermore, several authors treated patients with severe iliopsoas haematoma secondary to anticoagulant therapy with TAE [1,8,9]. This treatment proved to be successful and safe, especially for patients with surgical risk factors.

In the present case, given the effectiveness, safety and minimally invasive approach, we decided to submit the patient to TAE of the bleeding lumbar arteries. Indeed that patient responded very well to this treatment, both clinically and at CT evaluation. Also, platelet count and haemoglobin values progressively improved to normal values.

In conclusion, patients in treatment with heparin should be closely monitored for development of groin pain or femoral nerve palsy. Although very rare, bilateral iliopsoas haematomas, even if pT, INR and APTT values are within therapeutic range, can occur in otherwise healthy patients undergoing anticoagulant therapy. Early recognition of the bleeding by means of a CT scan is crucial to improving morbidity and mortality [1]. Once the diagnosis is made, further angiographic evaluation is recommended not only to detect any active bleeding, but also to perform TAE. Indeed, embolization of severely bleeding lumbar arteries avoids submitting the patient to surgical decompression of the haematomas, and prevents possible complications.

## Abbreviations

APTT: activated partial thromboplastin time
CT: computed tomography
INR: international normalised ratio
MRI: Magnetic Resonance Imaging
pT: prothrombin time
TAE: trans-catheter arterial embolization
